# Prostaglandin-Induced Cystoid Macular Edema Following Routine Cataract Extraction

**DOI:** 10.1155/2010/690707

**Published:** 2010-11-07

**Authors:** Negin Agange, Sameh Mosaed

**Affiliations:** Department of Ophthalmology, University of California, Irvine, CA 92697, USA

## Abstract

To our knowledge, we are reporting the first case of a 59-year-old man who developed recurrent CME with three separate trials of three different prostaglandin class drugs following uncomplicated phacoemulsification with intraocular lens implantation. Despite multiple reports of individual prostaglandin (PG) analogues being suggested as the cause of CME, there are no recommendations regarding withholding these medications in the perioperative period. Our patient first developed CME OD 4-months post uncomplicated cataract extraction. XALATAN (Latanoprost) had been restarted after surgery and discontinued at onset of CME. While off XALATAN (Latanoprost), the patient's CME resolved, but his IOP rose. The patient was started on LUMIGAN (Bimatoprost) to control the IOP, but within weeks his CME recurred. The patient's CME was again treated and his IOP remained acceptable, but then progressively increased. TRAVATAN (Travoprost) was attempted, but he presented with a third round of CME. Definitive conclusions about causal relationships cannot be made without well-designed, prospective clinical trials addressing this issue.

## 1. Introduction

The National Eye Institute estimates that there are 1.5 million surgeries for cataracts each year in the U.S. A common clinical scenario in a percentage of patients undergoing cataract extraction is receiving treatment for co-morbidities like glaucoma with hypotensive lipids. We report a case of a 59-year-old man who developed recurrent cystoid macular edema (CME) with three separate trials of prostaglandin analogs following uncomplicated phacoemulsification with intraocular lens implantation. Currently, we have no prospective, blinded-control trials to better define the ideal perioperative strategies in this patient population. 

## 2. Case Report

Our patient has a history of advanced pigmentary glaucoma in both eyes, which was controlled preoperatively for many years with XALATAN (Latanoprost), COSOPT (Timolol/Dorzolamide) fixed combination, and ALPHAGAN (Brimonidine) 0.1% purite OU. The patient developed visually significant cataracts in both eyes with visual acuity dropping below 20/40 OU prior to surgery. He had uncomplicated cataract extraction with IOL implant OD, followed one month later by OS. His best corrected visual acuity in each eye was 20/20 OU by one week post op. His immediate post-op regimen was AVELOX (Moxifloxacin) QID for one week, diclofenac QID for one month, and PRED FORTE (prednisolone) 1% QID for one week and then tapered off over the next three weeks. His XALATAN (Latanoprost) was resumed immediately post-op.

Our patient first developed CME in his right eye 4-months post-cataract extraction, when he presented with complaints of blurry vision and central distortion. A dilated fundus exam revealed cystoid macula edema, confirmed by OTC. The BCVA had dropped from 20/20 to 20/70. XALATAN (Latanoprost) was discontinued, and diclofenac was initiated. After 1-month, his BCVA and intraocular pressure OD were 20/70 and 16 mm Hg, respectively. Over the next two months while off the XALATAN (Latanoprost), the patient's CME improved, and visual acuity improved to 20/30 +2, but intraocular pressure climbed to 28 mmHg. Within three weeks of initiating LUMIGAN (Bimatoprost) in an effort to control his increased IOP his CME symptoms recurred for a second time, and his BCVA dropped again to 20/60. The patient's CME was again re-treated with a combination of topical diclofenac QID, and PRED FORTE (prednisolone) 1% QID. Prostaglandin therapy was avoided for a period of 13-months. His BCVA after resolution of the second episode of CME was 20/25 and remained stable for 13 months.

For one year after the second episode of CME, the IOP remained in the borderline acceptable range on ALPHAGAN (Brimonidine) 0.1% purite and COSOPT (Timolol/Dorzolamide) fixed combination OU. He became progressively worse when he presented with an IOP of 28 mmHg, at which time a trial of TRAVATAN (Travoprost) was attempted. Within 3-weeks the patient presented with third round of CME in the right eye. TRAVATAN (Travoprost) was the third prostaglandin therapy to date that seems to have triggered CME in our pseudophakic patient and was again discontinued. The patient's third episode of CME eventually resolved with the prior treatments, and his BCVA stabilized at 20/25. His IOP at the time of this report is controlled with COSOPT (Timolol/Dorzolamide) fixed combination and ALPHAGAN (Brimonidine) 0.1% purite.

## 3. Discussion

CME is a painless condition in which swelling or thickening occurs of the central retina (macula) and is usually associated with blurred or distorted vision. The primary cause of CME depends on the underlying disease process, but most pathways eventually lead to vascular instability and breakdown of the blood-retinal barrier. The Müller cells in the retina become overwhelmed with fluid leading to their lysis. This results in an accumulation of fluid in the outer plexiform and inner nuclear layers of the retina [1].

Endogenous prostaglandins (PGs) are known to modulate normal cell function as well as inflammatory response. The conventional route of aqueous humor flow is through the trabecular meshwork, Schlemm's canal and episcleral vessels. It is thought that PGs may regulate uveoscleral outflow by metalloproteinases- (MMP)-mediated alterations in the ciliary muscle in extracellular matrix metabolism [2–4]. It has also been shown that XALATAN (Latanoprost) induces cystoid macular edema (CME) in patients in the early postoperative period of cataract surgery as well as numerous case reports documenting CME with XALATAN (Latanoprost) use [5–7]. Halpern and Pasquale documents thirty-four eyes of thirty-two patients with documented CME in aphakia and pseudophakia [8].

Structurally, XALATAN (Latanoprost), TRAVATAN (Travoprost), and LUMIGAN (Bimatoprost) are all compounds related to prostaglandin 2*α*. Both XALATAN (Latanoprost) and TRAVATAN (Travoprost) are pharmacologically classified as prostaglandin analogs, but LUMIGAN (Bimatoprost) is considered to be a prostamide because it is an amide rather than an ester compound [9]. XALATAN (Latanoprost) is an isopropyl ester prodrug of 17-phenyl substituted prostaglandin F2*α* that effectively lowers IOP by enhancing uveoscleral outflow without significantly affecting other parameters of aqueous humor dynamics [10]. All drugs showed increased uveoscleral outflow, although the amount varied among the techniques [11].

However, the association between ocular hypotensive lipids and cystoid macula edema continues to be subject of debate. It has been suggested that it is unlikely topical XALATAN (Latanoprost) induces cystoid macula edema in eyes with a normally functioning blood-ocular barrier [5]. Interestingly, our patient, who had been uneventfully treated with XALATAN (Latanoprost) for several years, developed cystoid macular edema that worsened with rechallenge of LUMIGAN (Bimatoprost) and TRAVATAN (Travoprost), even more than one year after uncomplicated anterior segment surgery. Cystoid macular edema associated with XALATAN (Latanoprost) has been reported after uncomplicated cataract surgery, but it is an uncommon complication. In one retrospective review of a cohort of patients after uneventful phacoemulsification, 3% developed clinically significant CME [12]. It has also been associated with LUMIGAN (Bimatoprost) in an eye with high-risk profile. To our knowledge, this case represents the only report in the literature that definitively identifies prostaglandin analogs as the inciting agent in recurrent CME.

Optical coherence tomography (OCT) is helpful in establishing a diagnosis and in measuring the therapeutic response. Classically, patients with CME demonstrate retinal thickening and cystic spaces on OTC. Medical treatment modalities include corticosteroids, which directly inhibit the enzyme phospholipase, blocking the formation of prostaglandins. They are considered the primary treatment of CME in many instances, specifically in the treatment of CME secondary to uveitis. However, corticosteroids have many systemic and ocular adverse effects, and some patients become intolerant to them as a result. NSAIDs, like Diclofenac (Voltaren), inhibit the enzyme cyclooxygenase and can be used in the prevention and treatment of CME. NSAIDs are sually administered topically for approximately 3-4 months and on an asneededbasis. In a prospective, double-masked, study of indomethacin versus placebo in the treatment of patients undergoing cataract extraction the incidence of angiographic CME was higher in the placebo treated group [13]. In addition, treatment of CME with steroid and NSAIDs has been shown to be better than NSAIDs alone [14].

Despite multiple case reports of individual prostaglandin analogues being suggested as the cause of CME, there is currently no consensus or recommendation regarding the use of these medications in the perioperative period. Remissions and exacerbations of macular edema can result in photoreceptor damage with permanent impairment of vision. Definitive conclusions about causal relationships cannot be made without well-designed, prospective clinical trials addressing this issue given the large number of patients undergoing cataract extraction every year in the U.S. while being treated for glaucoma. More research in this area is important to understand if a causal relationship between the use of PG analogs and the occurrence of CME is more than anecdotal associations in the case reports. Caution might be advised when using PG analogs in eyes with risk factors for the development of CME.
